# On the Formation of Dentine

**Published:** 1877-11

**Authors:** Chr. Sihler


// THE DENTAL REGISTER.

Vol. XXXI.] NOVEMBER, 1877. [No. 11.

### ON THE FORMATION OF DENTINE.

BY CHR. SIHLER.

*Fellow, John Hopkins University, Baltimore, Md.*

When we inquire into the formation of dentine, we must take into consideration, 1. The fully formed dentine, its homogeneous substance infiltrated with limesalts; its main tubules and the side branches; the germinal matter which both the main and side tubes contains. 2. That the pulp, with its fibrous tissue, and blood-vessels and germs, disappears, and that we see dentine take its place. 3. We must take into consideration what is found where we make a section through the developing dentine and the soft tissue adhering to it, *i. e.*, when we examine growing teeth.

If we then remember that in such sections there is fully formed dentine, adjacent, a stratum of uncalcified texture, then the odontoblasts with their nucleus always on the base, *i. e.*, towards the pulp; that the odontoblasts are in continuity with the dentine by their main prosesses; that the odontoblasts have side fibres, (apparently), and are connected with each other laterally; that the direction of the fibres of the pulp is often at right angles to the direction of the dentinal canals, certainly never in any correlation with that of the dentinal tubes—when we further consider the want of union between the pulp and the dentine—then it seems to me the most probable conclusion is that the odontoblasts, or the vital processes occurring in them and through them, are the active agents in the production and formation of dentine, and that the pulp, with its vessels, germs and fibres, plays only a secondary role.

Supposing, then, we have before us as described, a certain thickness of finished dentine, the uncalcified stratum, the layer of odontoblasts, the pulp—what is now going on in the living tooth? how is the dentine increased in thickness? I think the nucleus of the odontoblasts is the part where vital action is the most intense, here the food, consisting of material carried there by the vessels of the pulp, and consisting of the adjacent parts of the pulp itself, is converted into germinal matter, while in the body of the odontoblast and the interior of the dentinal tubule conversion of the germinal matter into the formed matter is taking place. As the bioplast thus, in its anterior portion, absorbs the pulp, it spins the tubule in its wake, and in moving on secretes at the same time that it forms the tubules, the intertubular homogeneous matrix; after this is dense enough, limesalts are deposited in it, not in the way they are deposited in cartilage that is forming a granular appearance, but by uniting most intimately with the organic matrix and so forming almost a unit. While the odontoblasts are yet laterally in contact with each other, the side tubes are undoubtedly beginning to form as the glue between them is secreted, and as the odontoblast moves on they are further developed, and at the same time the direction which we perceive them to have, namely, always one of an acute angle between the outer extremity of the main tube and the side tube. That the side tubes are produced when the odontoblasts are yet free, is further proved by the processes which the odontoblasts often exhibit.

The view here given on the growth of dentine agrees, in the main, with that of Salter and Koelliker, only Salter does not mention the side tubes at all, and Koelliker says: "The finer processes of the dental fibrils are not present during the first formation, and have to be considered as secondary formations like the bone cells."

How this secondary formation shall take place is a matter of difficulty for my understanding; in fact, Koelliker does not aim to make it plausible. If Koelliker had taken into consideration that the odontoblasts, when attached to the dentine in the pulp cavity, are in contact laterally and united with each other; and further, that fibres, (apparently), are often seen standing out from their sides, and that a fibre and tubule cannot be distinguished by merely looking at them; and that the tubules appear just as soon as the limesalts are deposited; and that a thing may look homogeneous and yet not be so, he would have probably come to a more simple explanation.

That a tissue may appear homogeneous and yet may have tubules, or fibres, imbedded in it, can be especially and plainly seen in the recently formed layer of cementum; such, while yet uncalcified, looks perfectly homogeneous, just like cartilage, but upon the application of strong muriatic acid, which dissolves the homogeneous glue-like substance, a network of fibres or the network of canaliculi becomes apparent, which afterwards would have made their appearence as soon as the limesalts were deposited. It is well known amongst surgeons that the plaster dressing will shrink down when it dries. I think when the limesalts unite with the glue-like cement, dentine or bone or cementum, a little shrinkage may take place, and thus the tubules which it contains become more apparent.

The disappearance of the pulp, this theory explains by the action of the nucleus of the odontoblasts, and we have any amount of analogous processes in the absorption of deciduous teeth, bone cartilage, etc, as we will see hereafter when the formation of bone is discussed.

Quite different from the view here presented on the formation of dentine, is that of Waldeyer (and Tomes.) Waldeyer says, *Stricker's Manual*, page 337: "After it (the tooth papilla) has attained a certain size, the odontoblasts above described develop from the cells lying at the periphery, and we soon recognize a solid shell of dentinal bone superimposed on the dentine germ like a cap. The histological formation of the dentine is precisely similar to the ordinary process of ossification.

"Whilst the peripheric portion of the odontoblasts constantly undergo metamorphosis, with disappearance of their nucleus into a gelatinous matrix which subsequently undergoes calcification, their centric portions penetrate the hardened masses in the form of longer or shorter threads, and represent the first rudiment of the dental fibres. The lateral processes of the odontoblasts occasion the numerous anartomoses of the dental fibres, or of the dental tubule. Every odontoblast communicates with the nerve deeply situated, and successively enlarging cells of the young pulp by means of its pulp process, so that when an odontoblast is calcified up to the base of the fibre, another occurs in its place without any interruption of the continuity of the fibre. Hence, every dental fibre with its anastomoses must be regarded as formed of several continuous odontoblasts. The layers of matrix immediately surrounding the fibres undergo conversion, as appears from their chemical characters, into elastic tissue and form the dental sheaths of Neumann. It has not been ascertained whether they also undergo calcification. Thus it appears that the dentine, with all its constituents, proceeds from odontoblasts that have become metamorphosed in their form and chemical composition."

To understand the above one must read the description of the odontoblasts which Waldeyer gives: These cells (odontoblasts) are from 20 to 30mir. on the average in length, and about 5mir. in breath. They are fully granular and destitute of a membrane. The moderately large rounded or ovoid nucleus is usually contained in that end which is towards the pulp. Three kinds of processes may be distinguished in these cells: The dentinal processes, the pulp processes, and the lateral processes. The dentinal processes constitute the dentine fibres above described. It need here only be repeated that from one cell several dentine fibres are frequently given off. Such odontoblasts with several dentinal processes, are broad at the end directed toward the dentine, but as the processes pass on they gradually diminish to form dentinal fibres. The odontoblasts are intimately connected with each other by means of the five short teeth, which the lateral processes of all dentinal cells form. The short pulp process usually springs from the cell with a moderately broad base, and is constantly connected with one of the cells lying immediately beneath the membrane eloris, which last are usually much larger and more darkly granular than those more deeply seated.

Although such questions of formation and change will probably always be more or less matter of opinion; still I have to make some objections against the description of this process of dentine formation as offered by Waldeyer.

1. If Waldeyer would have described the formation of vascular dentine, then I could subscribe to his view; but supposing his description would explain the fully formed dentine, (which I think it does not), then the question would still be unanswered; how do we get rid of the vessels in the pulp that are so abundant? And before the side processes of the odontoblasts can be used there must be a conversion first of the fibres of the pulp tissue into the tubules of the dentine. Such questions must be at least touched.

2. I fail to see that the facts of the case are taken into consideration. How is it that the dentine separates so easily from the pulp? If there need only take place an infiltration of lime salts between the preformed tubules, then why this liability to separate? Or why do the fibres in the pulp take a course often at right angles with the dentinal tubules? And why are the germs in the pulp arranged in the same manner? Where is there any evidence for the disappearance of the nucleus, etc? The pulp is a real something, and not only an accumulation of "cells," for the benefit of histologists. It has to be got rid of. Is it an explanation to say that the matrix surrounding the fibres undergoes conversion into the sheath of Neumann?

3. Waldeyer exhibits four odontoblasts; all of them have a pulp process. I am somewhat at loss to understand how it comes that Waldeyer presents these four as typical ones; never have I, to my recollection, seen any such at all. Mine always had a truncated appearance towards the pulp side, and the nucleus at the end. I cannot, of course, deny that such exist as Waldeyer figures, but if they are typical ones, then the large number that I have seen must be the exceptions. I think the same argument holds good against the views of Tomes on the formation of dentine.

